# Effectiveness of a Smartphone App With a Wearable Activity Tracker in Preventing the Recurrence of Mood Disorders: Prospective Case-Control Study

**DOI:** 10.2196/21283

**Published:** 2020-08-05

**Authors:** Chul-Hyun Cho, Taek Lee, Jung-Been Lee, Ju Yeon Seo, Hee-Jung Jee, Serhim Son, Hyonggin An, Leen Kim, Heon-Jeong Lee

**Affiliations:** 1 Department of Psychiatry College of Medicine Chungnam National University Daejeon Republic of Korea; 2 Department of Psychiatry Chungnam National University Sejong Hospital Sejong Republic of Korea; 3 Korea University Chronobiology Institute Seoul Republic of Korea; 4 Department of Convergence Security Engineering College of Knowledge-based Services Engineering Sungshin Women's University Seoul Republic of Korea; 5 Department of Computer Science Korea University College of Information Seoul Republic of Korea; 6 Department of Psychiatry Korea University College of Medicine Seoul Republic of Korea; 7 Department of Biostatistics Korea University College of Medicine Seoul Republic of Korea

**Keywords:** circadian rhythm, digital therapeutics, mood disorder, recurrence prevention, smartphone app, wearable activity tracker

## Abstract

**Background:**

Smartphones and wearable devices can be used to obtain diverse daily log data related to circadian rhythms. For patients with mood disorders, giving feedback via a smartphone app with appropriate behavioral correction guides could play an important therapeutic role in the real world.

**Objective:**

We aimed to evaluate the effectiveness of a smartphone app named Circadian Rhythm for Mood (CRM), which was developed to prevent mood episodes based on a machine learning algorithm that uses passive digital phenotype data of circadian rhythm behaviors obtained with a wearable activity tracker. The feedback intervention for the CRM app consisted of a trend report of mood prediction, H-score feedback with behavioral guidance, and an alert system triggered when trending toward a high-risk state.

**Methods:**

In total, 73 patients with a major mood disorder were recruited and allocated in a nonrandomized fashion into 2 groups: the CRM group (14 patients) and the non-CRM group (59 patients). After the data qualification process, 10 subjects in the CRM group and 33 subjects in the non-CRM group were evaluated over 12 months. Both groups were treated in a similar manner. Patients took their usual medications, wore a wrist-worn activity tracker, and checked their eMoodChart daily. Patients in the CRM group were provided with daily feedback on their mood prediction and health scores based on the algorithm. For the CRM group, warning alerts were given when irregular life patterns were observed. However, these alerts were not given to patients in the non-CRM group. Every 3 months, mood episodes that had occurred in the previous 3 months were assessed based on the completed daily eMoodChart for both groups. The clinical course and prognosis, including mood episodes, were evaluated via face-to-face interviews based on the completed daily eMoodChart. For a 1-year prospective period, the number and duration of mood episodes were compared between the CRM and non-CRM groups using a generalized linear model.

**Results:**

The CRM group had 96.7% fewer total depressive episodes (n/year; exp β=0.033, *P*=.03), 99.5% shorter depressive episodes (total; exp β=0.005, *P*<.001), 96.1% shorter manic or hypomanic episodes (exp β=0.039, *P*<.001), 97.4% fewer total mood episodes (exp β=0.026, *P*=.008), and 98.9% shorter mood episodes (total; exp β=0.011, *P*<.001) than the non-CRM group. Positive changes in health behaviors due to the alerts and in wearable device adherence rates were observed in the CRM group.

**Conclusions:**

The CRM app with a wearable activity tracker was found to be effective in preventing and reducing the recurrence of mood disorders, improving prognosis, and promoting better health behaviors. Patients appeared to develop a regular habit of using the CRM app.

**Trial Registration:**

ClinicalTrials.gov NCT03088657; https://clinicaltrials.gov/ct2/show/NCT03088657

## Introduction

Mood disorders, including major depressive disorder and bipolar disorder, are prevalent throughout a person's life [1,2]. Patients with a mood disorder display a number of clinical manifestations such as mood lability, insomnia or hypersomnia, and reduced quality of life. Recently, there has been growing interest in the management of mood disorders as critical factors directly related to economic and social burden [3-5]. Patients with a mood disorder experience recurrent mood episodes, which are an important factor in determining the clinical prognosis and the chronic course [6,7]. Therefore, effective treatment of mood disorders is essential not only for acute treatment but also for preventing recurrence to enhance prognosis.

With the growth of digital health–related technology, there has been a renewed interest in apps related to mental health [8]. In particular, using daily log data obtained through various technologies called digital phenotypes, it is possible to acquire biometric data such as the amount of daily activity, mood state, sleep, and heart rate [9,10]. Various wearable devices and smartphone apps have already been investigated regarding their clinical applicability in psychiatric fields [11,12]. These new technologies are now being applied to the psychiatric field to determine whether they can play an active interventional role in the prevention, treatment, and prognosis management of psychiatric disorders [13,14]. In recent years, the concept of digital therapeutics has emerged, and attempts have been made to maximize the therapeutic effect by using these therapeutics instead of or in combination with conventional therapy [15]. Digital therapeutics can be considered a more suitable model for psychiatric and chronic diseases because they can help change daily habits or correct cognition and behavior. The advent of appropriate lifestyle-related treatment methods can contribute to better insights and health-related behavior. Until now, only biological and psychotherapeutic treatment have been used within limited clinical boundaries. Therefore, we can expect that in the future, mental health–related digital apps will help to combat these limitations based on real world evidence.

Circadian rhythm is reported to be closely related to clinical manifestations and the pathophysiology of mood disorders [16-19]. The disturbance of the circadian rhythm has been suggested as causing or resulting from the recurrence of a mood episode in patients with a mood disorder [20]. Therefore, measuring the disturbance of the circadian rhythm could reflect or predict the clinical deterioration of the mood disorder. Circadian rhythm can be measured at a biological level based on the expression of circadian genes and hormone secretion, as well as at a behavioral level based on sleep-wake, mealtime, or activity patterns, and the degree or timing of light exposure. With the development of digital technology and apps, smartphones and wearable devices can be used to obtain diverse daily log data related to circadian rhythms at a behavioral level [12,21]. We have reported a machine learning algorithm system using passive digital phenotypes of a wearable activity tracker and smartphone that can predict mood state or mood episodes by collecting daily log data from patients with a mood disorder [22]. For patients with a high risk of recurrence, giving feedback with appropriate behavioral correction guides could play an important therapeutic role in mood disorder management in the real world; this type of continuous feedback is not provided by conventional pharmacotherapy or psychotherapy.

In this study, we performed a 1-year prospective pilot study to investigate the effectiveness of the circadian rhythm of mood (CRM) app combined with a wearable activity tracker in preventing the recurrence of mood disorders. The primary hypothesis of this study is that the CRM app, which provides real-time mood episode risk prediction feedback through machine learning, will help to significantly reduce the recurrence of mood episodes in patients with a mood disorder.

## Methods

### Subjects and Study Design

The study was conducted between January 2017 and December 2018. The subjects were divided into the CRM and non-CRM groups. All patients met the criteria for major depressive disorder (MDD), bipolar disorder type I (BD I), or bipolar disorder type II (BD II) according to the Diagnostic and Statistical Manual of Mental Disorders, Fifth Edition [23]. For the CRM group, 14 patients (12 female, 2 male) diagnosed with a major mood disorder (MDD=1, BD I=11, and BD II=2) were recruited from the Korea University Anam Hospital. For the non-CRM group, 59 patients (30 female, 29 male) diagnosed with a major mood disorder (MDD=19, BD I=17, and BD II=23) were recruited from the Korea University Anam Hospital as a part of the Mood Disorder Cohort Research Consortium (MDCRC) study. The MDCRC study is a multicenter prospective observational cohort study investigating early-onset mood disorders in Korea, and its design and protocol have been reported previously [24]. Subjects with poor compliance were excluded from the analysis. Participants who had a wearable device wear rate (Wearable device wear rate = Total hours of wear/[365×24]) of <60% over the year were excluded. Finally, 43 patients (10 in the CRM group and 33 in the non-CRM group) were included.

Most of the study procedures related to gathering clinical symptoms and digital phenotypes were conducted in the same way in both groups, except for the daily feedback and warning alert in the CRM group. The recurrence of mood episodes was evaluated through regular follow-up visits based on the daily eMoodChart and confirmation by a psychiatrist (HJL or CHC) in both groups [24]. For the CRM group, information obtained in real time from each participant’s wearable device during the study period was analyzed using a circadian rhythm–based machine learning algorithm to predict the subject's current mood condition 3 days later [22].

The study was approved by the Institutional Review Board of Korea University Anam Hospital and conducted in accordance with the Declaration of Helsinki. All participants provided written informed consent before enrollment after receiving a full explanation of the study.

### eMoodChart and Wearable Device

All participants in both groups were requested to complete the daily eMoodChart and wear a wearable activity tracker (Fitbit Charge HR [2 or 3], Fitbit Inc) every day. The eMoodChart was a simple, intuitive assessment of their daily mood state (−3 to +3). Detailed information was mentioned in a previous report [24]. Subjects were asked to record their mood at 9 PM daily. If a patient missed a day, a reminder would be sent to their smartphone via SMS text messaging at 9 AM the next morning to remind them to record their previous day's mood by midday. This helped to minimize missing data and encouraged continuous recording.

For smartphones using the Android operating system, the built-in sensor could detect light exposure during the day and night. However, the light sensor data could not be collected for users with an iPhone due to Apple's restrictions. The activity trackers were worn on the wrist continuously and passively collected data related to activity, sleep, and heart rate. The data were then obtained by the researchers from the Fitbit cloud server.

### Circadian Rhythm for Mood (CRM) App

The feedback intervention for the CRM group consisted of three main functions: a trend report of mood prediction of the upcoming 3 days with facial icons, H-score feedback with intervention messages, and an alert system triggered if the overall H-scores were heading toward a high-risk state.

#### Trend Report of Mood Prediction

The purpose of the mood prediction feedback feature was to inform patients that their past behaviors can influence their future mood and to encourage them to change their behavior by showing the impact that these changes had on their mood. When the user turns the app on every day, the facial expression icon (emoticon) intuitively tells users the predicted result of how their mood will change over the next 3 days based on machine learning of real-time–acquired personal digital phenotypes (Multimedia Appendix 1). In the system, we used a mood prediction model evaluated in a previous study [22].

#### H-score Feedback With Intervention Messages

To provide feedback on changes in circadian rhythm, four types of H-scores were calculated for heart rate, activity, sleep, and light exposure. The following formula was used to calculate the H-score of the heart rate circadian rhythm (CR): CR_h_score = 0.5 N(CR_amplitude) + 0.5 (100 – N[CR_acrophase]). CR_amplitude refers to the amplitude of the circadian rhythm curve obtained when the cosinor method is applied to the heart rate data collected in the previous 48 hours [25,26]. CR_acrophase measures the distance between the midday reference point and the peak point of the currently observed rhythm curve. The function N(x) is a normalization function that converts x into a value between 0 and 100. The CR_h_score value is high when the height of the rhythm curve is getting higher and the rhythm is not misaligned (ie, not delayed or advanced). The following formula was used to calculate the activity rhythm H-score: ACT_h_score = 0.5 (100 – N[steps_during_bedtime]) + 0.5 N(steps_during_daytime). Steps_during_bedtime refers to the number of steps within the time zone bedtime, and steps_during_daytime means the number of steps within the time zone daytime. The following formula was used to calculate the light-exposed H-score: LE_h_score = 0.5 (100 – N[light_exposure_during_bedtime]) + 0.5 N(light_exposure_during_daytime), where light_exposure _during_bedtime and light_exposure_during_daytime refer to the average amount of light exposure at bedtime and during the daytime, respectively. The following formula was used to calculate the sleep H-score: SL_h_score = 0.5 N(sleep_efficiency) + 0.25 (100 – N[sleep_onset_dev]) + 0.25 (100 – N[sleep_offset_dev]). Sleep_efficiency is the ratio of the time spent sleeping without awakening to the total sleep time. Sleep_onset_dev represents the deviation between the ideal going-to-bed time (8 hours before sunrise) and the observed bedtime. Sleep_offset_dev is the deviation between the ideal and observed wake-up times. The weights of the components in each H-score calculation formula were set in a heuristic way.

Based on the four types of H-scores, various feedback such as a risk indicator of the H-score, a trend line of the changes in the H-score, and coaching messages are consequently generated on the CRM app (Multimedia Appendix 1). The risk indicator works according to the spectrum of the average of the four types of H-scores, and a trend line chart uses the average H-score. In the meantime, feedback messages are automatically generated depending on each type of H-score observation and reported on the app screen to help patients change their behavior by reducing bad habits and encouraging good habits toward their desirable H-score management.

#### Warning Alert for Irregular Life Patterns

If a patient had irregular life patterns, warning alerts were sent in addition to the feedback messages. The warning alerts were delivered to subjects when the average of the four H-scores fell below 50 or the average was below 60 for two consecutive days. Warning alerts were sent to patients via an SMS text message and read “Recently, your life rhythm is irregular.” It was hoped that the feedback messages and warning alerts would help prevent recurrences by helping the subject to recognize and modify their behaviors.

### Data Sets From Patients’ Smartphones and Wearable Devices

A total of 26,645 (36573) data points were collected from 73 patients (14 in the CRM group and 59 in the non-CRM group) over the 1-year study period. After excluding noncompliant subjects who had a wearable device wear rate of <60%, 15,695 (36543) data points were collected from a total of 43 patients (10 in the CRM group and 33 in the non-CRM group). To observe changes in circadian rhythm in the selected compliant group, representative data of (1) light exposure, (2) activity, (3) sleep, and (4) heart rate were automatically collected through smartphone and Fitbit devices. To capture the factors related to circadian rhythm from digital phenotypes, we extracted a total of 13 basic features, as in our previous study (Multimedia Appendix 2) [22]. Three types of periods (*n* values include 3, 6, and 12 days) and three representative values of the mean, standard deviation, and gradient of the trend line of feature values are extracted as additional features during the *n* days and reflected in the model training. In total, 130 features were used in model construction, including 13 basic features and 117 additionally extended features (13 basic features 3 types of periods 3 representative values for each period). The detailed criteria and methods have been reported previously [22].

### Assessments

#### Comparison of the Recurrence Rate Between the CRM Group and Non-CRM Group

During the 1-year study period, we compared the clinical progress of the two groups. The recurrence of mood episodes was evaluated by a psychiatrist (HJL or CHC). The number and duration of mood episodes were used as indicators, and the differences were compared by separating the depressive and manic/hypomanic episodes. Additionally, the number and duration of the total mood episodes were compared.

#### Effectiveness of Feedback Intervention of the CRM App on Behavioral Changes

To evaluate the effectiveness of the feedback system of the CRM app with regard to circadian rhythm, two aspects were evaluated. First, after patients received a warning alert, we observed whether this had a positive effect on their circadian rhythm-related features. Therefore, we tried to gauge the effect of the alert by observing what changes occurred in the features before and after the warning alert was received; the changes were observed by comparing the period 3 days before receiving the alert and 3 days after receiving the alert. For a time point *t* of a warning alert occurrence, given that two feature time series, the following equations are used: X_before_ = (x_t-3_, x_t-2_, x_t-1_, x_t_) from the period <t-3, t> and X_after_ = (x_t_, x_t+1_, x_t+2_, x_t+3_) from the period <t, t+3>, the measure *delta of gradient change* (DGC) is calculated using DGC = g(X_after_) - g(X_before_). Here, g(X) is a function that returns the slope value (gradient) of the trend line obtained through a linear regression analysis for a given time series data *X*. Thus, if the DGC value is positive, the feature *x* tends to increase after the warning alert, and if negative, it means that it tends to decrease. 

Second, after patients received feedback (eg, H-score graphs, emotional facial icons, warning alerts), we observed whether there was a positive change in relation to their active use of our proposed system. To quantitatively measure user loyalty in terms of active use, the wearable device wear rate of patients was investigated over time. The wear rate refers to the percentage of time that subjects wore their Fitbit over a month, that is, the wear rate is the total hours of wear divided by 720 hours (3024). Changes in the wear rate during the study period were calculated, and the results were compared between the CRM and non-CRM groups.

### Statistical Analyses

The demographic data and disease-related variables at baseline of the two groups were compared using the chi-square test, *t* test, or Fisher exact test, as appropriate. A generalized linear model (GLM) analysis was used to compare the number and duration of the total and individual mood episodes during the study period between the CRM and non-CRM groups. As the two groups were not matched samples, a GLM analysis was performed considering these limitations. In this process, the baseline variables that displayed significant differences were regarded as confounding variables and adjusted for the multivariable GLM. Analyses were performed using SAS 9.4 (SAS Institute Inc).

Kolmogorov-Smirnov and Mann-Whitney *U* tests were used to analyze whether there was a significant difference in the distribution of DGC values between the two groups using the Python SciPy tool. The Kolmogorov-Smirnov test was used to verify that the DGC outcomes from each of the two groups were from different distributions. The Mann-Whitney *U* test was used to show that the median difference in the DGC samples between the two groups was statistically significant.

## Results

### Participant Demographics and Clinical Information

For the CRM group (n=10), the average age (SD) of the patients, age at the first onset of mood disorder, and age at first psychiatric treatment were 35.30 years (SD 5.33), 16.40 years (SD 5.58), and 22.10 years (SD 9.87), respectively. Furthermore, for the non-CRM group (n=33), the average age of the patients, age at the first onset of mood disorder, and age at first psychiatric treatment was 22.97 years (2.86), 18.00 years (SD 4.81), and 20.42 years (SD 3.95), respectively. The demographic and baseline variables of the two groups are presented in Table 1.

There were statistically significant differences between the two groups in age (*P*<.001), previous psychiatric admission (*P*=.02), previous depressive episodes (*P*=.007), and previous manic episodes (*P*=.005). The following main analyses were conducted by correcting the baseline variables showing significant differences.

**Table 1:** Basic demographic and clinical information of the CRM group and non-CRM group.

Demographics and clinical information		Circadian rhythm for mood group (N=10)	Non–circadian rhythm for mood group (N=33)		*P* value
Gender (male), n (%)		2 (20)	15 (45)		.27^a^
Age (years), mean (SD)		35.30 (5.33)	22.97 (2.86)		<.001^b^
**Diagnosis, n (%)**				.07^c^	
	Bipolar disorder type I	8 (80)	12 (36)		N/A^d^
	Bipolar disorder type II	1 (10)	10 (30)		N/A
	Major depressive disorder	1 (10)	11 (33)		N/A
**Type of mood episode at the first onset, n (%)**				.68^c^	
	Depressive episode	8 (80)	29 (88)		N/A
	Manic episode	2 (20)	3 (9)		N/A
	Hypomanic episode	0 (0)	1 (3)		N/A
Age at first onset of mood disorder, mean (SD)		16.40 (5.58)	18.00 (4.81)		.38^e^
Age at the first visit to a psychiatric clinic, mean (SD)		22.10 (9.87)	20.42 (3.95)		.61^e^
Number of previous psychiatric administration, mean (SD)		2.10 (1.60)	0.97 (1.36)		.02^b^
Number of previous depressive episodes, mean (SD)		11.60 (5.42)	6.64 (7.45)		.007^b^
Age at the first onset of depressive episode, mean (SD)		16.13 (6.15)	17.45 (4.76)		.52^e^
Number of previous manic episodes, mean (SD)		3.90 (3.81)	0.82 (1.29)		.005^b^
Age at the first onset of manic episode, mean (SD)		17.50 (3.54)	22.67 (3.79)		.22^e^
Number of previous hypomanic episodes, mean (SD)		10.40 (12.36)	3.45 (6.00)		.06^b^
Age at the first onset of hypomanic episode, mean (SD)		N/A	N/A		N/A

^a^Chi-square test.

^b^Wilcoxon rank-sum test.

^c^Fisher exact test.

^d^N/A: not applicable.

^e^*t* test.

### Comparison of the Recurrence of Mood Episodes Between the CRM Group and Non-CRM Group

The univariable GLM analysis showed that the CRM group had 60.7% fewer total depressive episodes (n/year; exp β=0.393, *P*=.03), 48.5% shorter depressive episodes (total; days/year) (exp β=0.515, *P*<.001), 85.7% shorter manic/hypomanic episodes (days/year; exp β=0.143, *P*<.001), 66.4% fewer total mood episodes (exp β=0.336, *P*=.01), and 63% shorter mood episodes (total; exp β=0.370, *P*<.001) than the non-CRM group (Table 2).

The multivariable GLM was applied after adjusting for gender, age, distribution of mood disorders, mood episode type at the first onset, age at the first onset, age of first psychiatric visit, number of previous psychiatric admissions, number of depressive episodes, number of manic episodes, and number of hypomanic episodes. The multivariable GLM analysis showed that the CRM group had 96.7% fewer total depressive episodes (n/year; exp β=0.033, *P*=.03), 99.5% shorter depressive episodes (total; exp β=0.005, *P*<.001), 96.1% shorter manic/hypomanic episodes (exp β=0.039, *P*<.001), 97.4% fewer total mood episodes (exp β=0.026, *P*=.008), and 98.9% shorter mood episodes (total; exp β=0.011, *P*<.001) than the non-CRM group (Table 2).

**Table 2:** Comparison of the recurrence of mood episodes between the circadian rhythm for mood group (n=10) and the non–circadian rhythm for mood group (n=33).

Mood episodes	Univariable generalized linear model		Multivariable generalized linear model	
	Exp β (95% CI)	*P* value	Exp β (95% CI)	*P* value
Total depressive episodes (n/year)	0.393 (0.16-0.99)	.048	0.033 (0.00-0.71)	.03
Major depressive episodes (n/year)	0.532 (0.21-1.37)	.191	0.347 (0.00-1.19)	.06
Minor depressive episodes (n/year)	N/A^a^	N/A	N/A	N/A
Brief depressive episodes (n/year)	N/A	N/A	N/A	N/A
Duration of total depressive episodes (days/year)	0.515 (0.45-0.59)	<.001	0.005 (0.00-0.01)	<.001
Total manic/hypomanic episodes (n/year)	0.388 (0.09-1.68)	.21	0.005 (0.00-1.74)	.08
Manic episodes (n/year)	N/A	N/A	N/A	N/A
Hypomanic episodes (n/year)	0.550 (0.12-2.46)	.43	0.005 (0.00-17.22)	.20
Duration of manic/hypomanic episodes (days/year)	0.143 (0.10-0.21)	<.001	0.039 (0.02-0.08)	<.001
Total mood episodes (n/year)	0.336 (0.14-0.78)	.01	0.026 (0.00-0.38)	.008
Duration of total mood episodes (days/year)	0.370 (0.32-0.42)	<.001	0.011 (0.01-0.02)	<.001

^a^N/A: not applicable.

### Effectiveness of the Feedback System in Terms of Behavioral Changes

**Figure 1:**
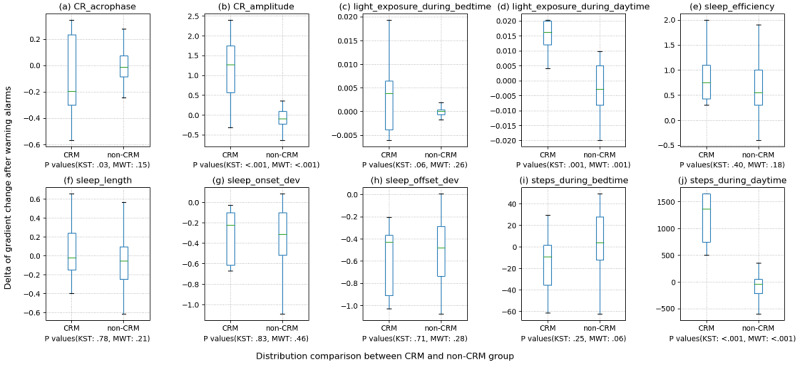
Comparison of the distributions of changes in individual features after receiving warning alerts in the CRM and non-CRM groups. The vertical axis refers to the delta of gradient change (DGC). If the median of the box plot (the green horizontal line inside the box) is near zero, this means that there was no significant change in the feature before and after the warning alert. If the median is greater than zero, this means that the feature value tended to increase after the alert. If it is less than zero, this means that the feature value tended to decrease after the alert. For example, if a patient with low activity during the day received a warning alert and the median of the distribution of the DGC value outcome for the feature steps_during_daytime moves in a positive direction, then we can assume that the patient tried to increase their activity. To compare the DGC distributions of the CRM group and non-CRM group for key features related to the H-score, DGCs were calculated based on each of the time points t at which alarms were activated during the experiment. The system logs for the warning alert calculations recorded the past feedback point t of the alert receivers. The actual warning alert was not delivered to the non-CRM group at time t but delivered only to the subjects in the CRM group at time t. In the comparison analysis, patients whose features could not be calculated due to a lack of data were omitted. CRM: Circadian Rhythm for Mood; KST: Kolmogorov-Smirnov test; MWT: Mann-Whitney *U* test.

The distribution of the DGC results between the CRM and non-CRM groups can be seen in Figure 1. To compare the results between the two groups, statistical verification was performed for the median DGC values from individuals. Consequently, significant positive behavioral changes after receiving warning alert feedback (assuming 95% CIs, *P*<.05) were CR_amplitude, light_exposure_during_daytime, and steps_during_daytime. In the light_exposure_during_daytime and steps_during_daytime features, an increasing tendency of DGC was observed, meaning that CR_amplitude had increased. Most of the advice provided in the warning alert was about increasing the amount of activity. However, regarding sleep, although mean differences in DGC apparently existed between the two groups, statistical significance was not observed. We found that the efficiency and duration of sleep tended to increase after the warning alert point in both groups (in the non-CRM group, this does not correspond to a real warning alert point). This may be because people naturally replenish their sleep the next day after experiencing trouble sleeping (eg, insomnia experience).

**Figure 2:**
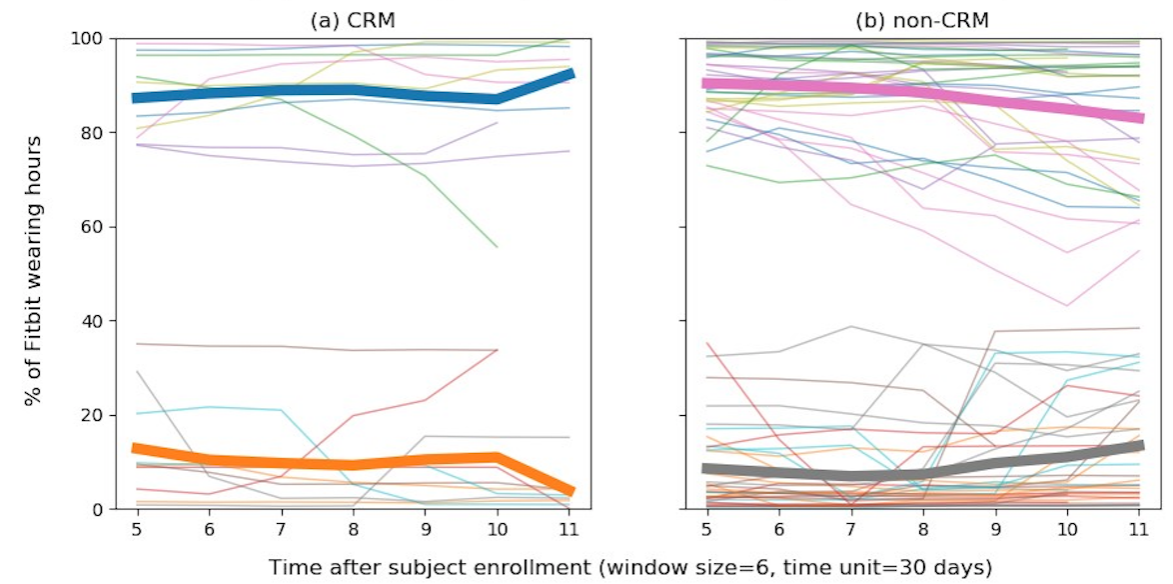
Changes in the Fitbit wearing rate in the CRM and non-CRM groups. A moving average technique was used to report the trend lines by abbreviating the raw time series data (window size=6 days). The time unit on the horizontal axis is 30 days, which means that the number of total wearing hours for 30 days was measured and plotted at each time point. The thin solid lines on the top area are individual trend lines of the change in the Fitbit wearing rate of each patient, and the thick solid line is an average trend line of the individual lines. The lines in the bottom area are the standard deviation trend line (MSD) of the change in the wearing rate. Increasing moving average and decreasing MSD might be positive.

The change in Fitbit wearing rates can be seen in Figure 2. Positive results (ie, maintenance of the wearing rate) were observed in the CRM group but not in the non-CRM group. The trend lines for changes in Fitbit wearing rates were analyzed in two aspects: the moving average (MA) and moving standard deviation (MSD). In the CRM group, the average MA trend line for the wearing rate was almost static over time and increased in the last 60 days. The average MSD trend line remained horizontal and maintained a stably low value without any significant change. This means that the variation in wearing rates was not significant over time in the CRM group. Contrastingly, in the non-CRM group, the average MA trend line shows a downward curve over time, meaning that the Fitbit wearing rate gradually decreased compared to that in the CRM group. Regarding the average MSD trend line, the upward curve shows that the wearing rate in the non-CRM group did not remain stable over time and that the deviation gradually increased.

## Discussion

### Principal Findings

In this study, we implemented a mood prediction algorithm using a passive digital phenotype in patients with mood disorders previously developed [22] as a CRM feedback system, and conducted a prospective pilot study for 1 year to see if it is effective in preventing the recurrence of mood episodes. We confirmed that giving daily circadian rhythm–based mood prediction feedback by analyzing the life patterns of individual patients with mood disorders significantly lowers the number and duration of mood episodes compared to those in the control group.

As mood disorders are recurrent and progress to a chronic course, reducing the recurrence of mood episodes and improving the prognosis is key to treatment [27,28]. In this regard, it is particularly important to track the real-time psychiatric condition, evaluate the risk of recurrence, and feed it back to help the patient identify the personal characteristics of their mood disorder. Previously, efforts have been made to maintain a stable euthymic state through conventional treatment, including pharmacotherapy [29,30], and there have been reports that nonpharmacological treatments such as cognitive behavioral therapy (CBT) or interpersonal social rhythm therapy can help manage prognosis [31,32]. This study observed the prognosis for 1 year depending on whether the CRM app feedback was applied while maintaining conventional treatment, and a strong and noticeable recurrence prevention effect was observed. The CRM app could be classified as a nonpharmacological treatment, but it appears to be more effective compared to the existing nonpharmacological treatment methods. Considering the study period and the number of samples, the results related to the number of mood episode recurrences should be considered. However, when comparing the periods of depressive, manic/hypomanic, and overall mood episodes, all were significantly shorter than 90% of those in the non-CRM group, which is a favorable result. The markedly shortened period of mood episodes in the CRM group needs to be considered in terms of preventing the recurrence effect of the CRM app and shortening the recovery time after the recurrence of mood episodes. Shortening of the mood episode duration in case of relapse may be affected not only by prevention but also by resilience and speed of recovery. The CRM app provides a guide to mood prediction and life guidance based on circadian rhythms. In addition, it helps patients acquire relevant insights to help them establish regular circadian rhythm–based lifestyle patterns in their daily lives. It could be interpreted that the behavioral change and ongoing use of the CRM app can not only prevent recurrence but also help in rapid recovery after a relapse.

Most of the current conventional treatments have limitations. To overcome the time, space, and cost limitations of conventional treatment methods, the concept of digital therapeutics has recently emerged as a new therapeutic tool [33]. Most digital therapeutics are based on CBT and offer solutions to psychiatric problems in real life [15]. However, it is difficult to provide customized treatment solutions for each patient because the therapist is not leading and individually engaging in therapeutic interventions based on the patient's symptoms and situations through digital therapeutics. Therefore, until now, digital therapeutics with relatively standardized conventional psychotherapy methods such as CBT-I have been actively implemented [34,35]. However, digital therapeutics that provide standardized treatments rather than personalized therapeutic interventions do not provide an agile response to changes in patient symptoms and situations and may result in decreased compliance with increased user fatigue. In particular, in patients with a mood disorder, mood states are influenced by a variety of factors and change so much that standardized therapeutic interventions are difficult, and digital therapy tailored according to symptoms is exceedingly difficult. In this study, since individual mood prediction was applied using passive digital phenotypes of patients with mood disorders, this strategy represents a step forward compared to the digitization of existing standardized therapeutic tools. For the practical clinical application of psychiatric digital therapeutics, it is important to quickly and accurately identify and predict psychiatric conditions (such as mood) using digital phenotypes in real time, and to provide individual therapeutic feedback to patients who are real users.

In the field of psychiatry, the rapid evaluation and diagnosis of psychiatric conditions and prognosis management through timely therapeutic interventions are considered to be particularly important. Furthermore, treatment adherence is an important factor in determining prognosis [36,37]. Therefore, the application of digital technology to psychiatry should be directed toward improving treatment compliance and increasing the associated therapeutic value. In particular, predicting mood or recurrence in patients with a mood disorder would enable faster interventions. Previous studies have predicted mood using variables of multiplex dimensions. Attempts to predict mood or recurrence using traditional markers such as biological markers and brain imaging markers have been made; recently, an analysis using voice, natural language, and variables from wearable devices was undertaken [22,38-42]. In conducting a recurrence prediction study for these mood disorders, (1) predictive performance, (2) practical applicability, and (3) privacy protection should be considered. Prediction performance can increase accuracy if a precise method is applied using as many different layers of data as possible. However, the difficulty of acquiring or analyzing data is exceedingly difficult, and if this is too expensive or takes too long, then its practical applicability is reduced. Therefore, it is important to acquire real-time data at as low a cost as possible to improve predictive performance. Even so, personal variables such as personal health records, voice, location information, and natural language can cause various ethical and legal problems [43,44]. It can be said that this study satisfies the conditions mentioned above because only passive digital phenotypes (such as activity, heart rate, and sleep), which are difficult to identify, were utilized. Thus, our method not only has clinical value but also has practical applicability.

The biggest problem in using digital devices is ensuring that compliance and engagement remain high. In general, as the length of time a wearable device is used increases, the fatigue rate increases and the usage rate decreases [45,46]. In this study, the wear rate of the wearable device was evaluated separately between the two groups, and it was confirmed that the wear rate was continuously and stably maintained in the CRM group compared to that in the non-CRM group. This suggests that feedback of useful information to the user (patient) can help to keep the digital device usage rate stable and consistent. Many obstacles must be overcome for the actual clinical application of digital devices and therapeutics. However, there are concerns about technology development, but if the user does not use the developed ones, there is no benefit. In addition, since continuous long-term use is essential for prevention or prognosis management, it is a significant obstacle if the device is only used for a short period of time. To overcome this, there are attempts to provide financial compensation such as incentives or intervention by digital device or treatment managers to maintain compliance [45,47], but there are certain limitations to these tactics in terms of cost-effectiveness. This study shows that the provision of useful information and feedback to users may be vital in maintaining compliance, and it is necessary to develop a technology focused on this. Additionally, it could be speculated that continued compliance in this study may have contributed to the therapeutic effect. However, this is difficult to clarify.

In the H-score calculation, one of challenging issues was to determine an appropriate weight combination. The issue requires further research. In the H-score calculation, if more large-scale patient data can be collected through continuous research in the future, sensitivity analysis will find more appropriate weights mathematically. In this paper, the weight was set to have an equal ratio by recognizing the importance of equality rather than bias among the determinants when calculating H-score. However, there was one exception, and in the case of sleep H-scores, the effect of too little sleep was so great that appropriate weights were set accordingly.

The strengths of this study are as follows. First, the therapeutic effect was studied by implementing a feedback function of a circadian rhythm–based mood prediction algorithm using a digital phenotype verified through previous studies, and a strong recurrence prevention effect was confirmed. Second, although this study is a pilot study, the patients were followed for 1 year, which is sufficient for confirming whether a recurrence occurred. Due to the clinical characteristics of mood disorders, it is exceedingly difficult to observe recurrence. Third, although a sham app was not used, both groups were allowed to use the app and wearable device, thereby minimizing any differences in effect. Fourth, the effects were presented through integrated analysis such as behavior change and compliance as well as recurrence prevention.

### Limitations

There are several limitations to this study. First, the case-control sample was not sufficiently matched. Although this is a pilot study to investigate the effectiveness of the CRM app, the case-control groups were not matched samples. We tried to perform statistical analysis by applying the GLM analysis with statistical experts to overcome the sample imbalance. Interestingly, the number of previous psychiatric admissions, number of previous depressive episodes, and number of previous manic episodes were significantly higher in the CRM group in the clinical information analysis. Despite the relatively negative clinical history of the CRM group, it is noteworthy that the pilot study showed a strong positive effect. Second, since this study was not conducted as a randomized controlled trial, allocation to the CRM feedback intervention was nonrandomized. As a result, it is not possible to completely exclude factors such as the influence of the characteristics of patients using the CRM app in the interpretation of the research results. The research team plans to conduct a randomized controlled trial on matched samples to produce reliable research results. Third, the sham app corresponding to the CRM app feedback system was not completely provided to the control group. Fourth, light sensor data could not be collected from iPhone users for technical reasons, so this could not be analyzed.

### Conclusions

This study investigated the effectiveness of a CRM app with an activity tracker for recurrence prevention in patients with a major mood disorder. The total mood episodes were fewer and shorter in the CRM group than in the non-CRM group. In the CRM group, positive changes in health behavior due to the warning alerts and maintenance of wearable device adherence rates were observed. The CRM app with a wearable device was found to be effective in preventing and reducing the recurrence of mood disorders, improving prognosis, and promoting better health behavior. Patients appeared to develop a regular habit of using the CRM app. This study is valuable as a preliminary study confirming that the mood prediction machine learning algorithm and feedback system using a circadian rhythm–based digital phenotype are clinically effective and might be used as the basis for the digital treatment of mood disorders. In the future, the CRM app’s effectiveness should be verified using a randomized controlled trial with a larger number of patients.

## Appendix

Multimedia Appendix 1 Screen capture of the CRM app developed and used in the current study. CRM: circadian rhythm for mood.

Multimedia Appendix 2 The proposed basic features to check circadian rhythm from the automatically measured passive digital log data of patients with mood disorders.

## Abbreviations

CBT: cognitive behavioral therapy
CRM: circadian rhythm for mood
GLM: generalized linear model
HR: heart rate
MA: moving average
MDCRC: Mood Disorder Cohort Research Consortium
MDD: major depressive disorder
MSD: moving standard deviation
