# Supplementary material for: Effectiveness of a Smartphone App With a Wearable Activity Tracker in Preventing the Recurrence of Mood Disorders: Prospective Case-Control Study
Source: JMIR Ment Health. 2020 Aug 5;7(8):e21283. doi: 10.2196/21283 (PMC7439135; doi:10.2196/21283)
Supplement: Multimedia Appendix 1 [file mental_v7i8e21283_app1.docx]

## Multimedia Appendix 1

**Screen capture of the CRM application developed and used in the current study**

**
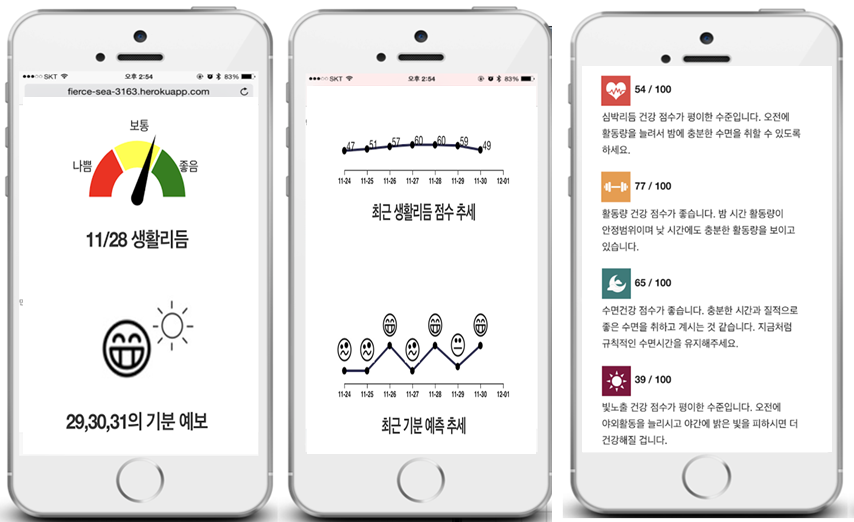
**
