# Supplementary material for: Effectiveness of a Smartphone App With a Wearable Activity Tracker in Preventing the Recurrence of Mood Disorders: Prospective Case-Control Study
Source: JMIR Ment Health. 2020 Aug 5;7(8):e21283. doi: 10.2196/21283 (PMC7439135; doi:10.2196/21283)
Supplement: Multimedia Appendix 2 [file mental_v7i8e21283_app2.docx]

## Multimedia Appendix 2

**The proposed basic features to check circadian rhythm from the automatically measured passive digital log data of patients with mood disorders**

| **Category** | **Feature name** | **Description** | **Rationale** |
| --- | --- | --- | --- |
| Light exposure | light_exposure_during_bedtime | The cumulative amount of light exposure during bedtime, which is the period 8 hours before sunrise to the time of sunrise. | Light is the most important trigger that synchronizes the circadian rhythm. Being exposed to light at night is not desirable but is recommended during the daytime. |
|  | light_exposure_during_daytime | The cumulative amount of light exposure during the daytime, which is the period between sunrise and sunset. |  |
| Step | steps_during_bedtime | The cumulative count of steps during bedtime | Sufficient walking is recommended during the daytime and walking should be avoided around bedtime for a sound circadian (sleep-wake) rhythm. |
|  | steps_during_daytime | The cumulative count of steps during the daytime |  |
| Sleep | sleep_length | The length of time asleep | Sufficient and regular sleep is important for achieving a sound circadian rhythm and relieving fatigue and stress. |
|  | sleep_quality | The quality score of sleep between 0 to 100. It is calculated by: (sleep length - restless sleep length) / sleep length |  |
|  | sleep_onset_dev | Deviation of sleep onset times is measured to check whether sleep onset times are regular. A deviation means that the observed time distances from the moment, 8 hours ago, before the sunrise time of the next day. |  |
|  | sleep_offset_dev | Deviation of sleep offset (wake-up) time is measured to check whether the sleep offset times are regular. Deviation means the observed time distance from the sunrise time. |  |
| Heart rate (HR) | CR_amplitude | The daily HR rhythm of the cosine curve as its amplitude. A large-amplitude means a clear rhythm curve with low HR in sleep and high HR in activity. | The HR contains important rhythmic information. HR falls when sleeping and goes up when activity increases. Therefore, it is ideal to have an S-shaped cosine curve in the HR graph as this shows that sleep is occurring at night and activity during the day. From the cosinor analysis for daily HR, four parameters were produced: amplitude, acrophase, mesor, and R-squared. |
|  | CR_acrophase | The daily HR rhythm of the cosine curve has a peak point at time t, where t is acrophase. Acrophase tells a degree of misalignment of rhythm. |  |
|  | CR_mesor | Mesor is the average HR per day. The more active HR, the higher the mesor. |  |
|  | CR_goodness_of_fit | R-squared means good fitness of the daily HR in the cosine curve fitting. The better the fit, the higher the R-squared. |  |
|  | resting_heartrate | The average HR within timeslots where a user has no activity (subject's resting). | Measuring HR at rest is ideal. It tends to rise when stressed, anxious, or unfit, which might be correlated with mood state. |
